# Protein Kinase C Epsilon and Genetic Networks in Osteosarcoma Metastasis

**DOI:** 10.3390/cancers5020372

**Published:** 2013-04-08

**Authors:** Atta Goudarzi, Nalan Gokgoz, Mona Gill, Dushanthi Pinnaduwage, Daniele Merico, Jay S. Wunder, Irene L. Andrulis

**Affiliations:** 1 Department of Molecular Genetics, University of Toronto, 1 King’s College Circle, Toronto, ON M5S 1A8, Canada; E-Mail: andrulis@lunenfeld.ca; 2 Samuel Lunenfeld Research Institute, Mount Sinai Hospital, 600 University Ave., Toronto, ON M5G 1X5, Canada; E-Mails: nalan@lunenfeld.ca (N.G.); monagill_@hotmail.com (M.G.); pinnad@lunenfeld.ca (D.P.); wunder@lunenfeld.ca (J.S.W.); 3 The Centre for Applied Genomics, The Hospital for Sick Children, MaRS Centre—East Tower, 101 College Street Rm.14-701, Toronto, ON M5G 1L7, Canada; E-Mail: daniele.merico@gmail.com

**Keywords:** osteosarcoma, metastasis, expression profiling, network analysis, Dynemo, protein kinase C epsilon

## Abstract

Osteosarcoma (OS) is the most common primary malignant tumor of the bone, and pulmonary metastasis is the most frequent cause of OS mortality. The aim of this study was to discover and characterize genetic networks differentially expressed in metastatic OS. Expression profiling of OS tumors, and subsequent supervised network analysis, was performed to discover genetic networks differentially activated or organized in metastatic OS compared to localized OS. Broad trends among the profiles of metastatic tumors include aberrant activity of intracellular organization and translation networks, as well as disorganization of metabolic networks. The differentially activated PRKCε-RASGRP3-GNB2 network, which interacts with the disorganized DLG2 hub, was also found to be differentially expressed among OS cell lines with differing metastatic capacity in xenograft models. PRKCε transcript was more abundant in some metastatic OS tumors; however the difference was not significant overall. In functional studies, PRKCε was not found to be involved in migration of M132 OS cells, but its protein expression was induced in M112 OS cells following IGF-1 stimulation.

## 1. Introduction

Osteosarcoma (OS) is the most common primary malignant cancer of the bone, with nearly 1,000 diagnoses annually in North America [1,2]. High-grade intramedullary osteosarcoma (“conventional”) constitutes the majority of cases, and is an aggressive disease that typically metastasizes to the lungs [1,2]. Approximately 10–20% of patients present to the clinic with discernable metastasis, and a further 20–30% develop metastasis despite aggressive treatment [3]. Currently neoadjuvant chemotherapy in combination with surgical resection of osteosarcomas can achieve 5-year overall survival rates of approximately 65% [4,5]. However, delineation of the molecular mechanisms contributing to osteosarcoma metastasis has the possibility of identifying specific therapeutic targets that improve prognosis of patients with metastatic osteosarcoma.

Expression profiling is an “omic”-level discovery technique that allows the quantification of thousands of different transcripts simultaneously between different disease states [6]. This technology has many applications in the cancer field including distinguishing subtypes of a particular cancer, identifying transcripts differentially expressed between those types, and predicting the subtype of a cancer based on the expression of identified transcripts [6,7]. In recent years it has been shown that analysis of expression profiles at the level of sets of transcripts rather than individual transcripts has more statistical power, is more reproducible between studies, and provides informative context to the results [7,8,9]. There are many algorithms for gene-set or network-based analytical algorithms, including the popular gene-set enrichment analysis (GSEA) and the proprietary ingenuity pathway analysis (IPA) [10]. Additionally dozens of other network-based analytical algorithms have been published that either utilize different statistical methods or that interrogate a unique cellular application (reviews: [8,9]). Two such algorithms are the methods of Chuang *et al.* that aims to discover differentially “activated” genetic networks [11], and “Dynemo” published by Taylor *et al*. that aims to discover differentially organized genetic networks [12]. These algorithms have successfully been applied to uncover molecular alterations in expression profiles of poor-outcome breast cancers, and have achieved over 70% predictive accuracy in -classifying those tumor samples utilizing a five-fold cross-validation strategy [11,12].

The aim of the present study was to identify differentially activated and organized networks in expression profiles of metastatic-at-diagnosis osteosarcomas (MD-OS) compared to expression profiles of localized-at-diagnosis osteosarcomas (LD-OS). This analysis would assist in the prioritization of candidate networks for *in vitro* characterization in osteosarcoma cell lines, including cells of differing metastatic capability.

## 2. Results

### 2.1. Unsupervised Hierarchical Clustering of Expression Profiles Reveals Distinct Subtypes of Osteosarcomas

**Figure 1:**
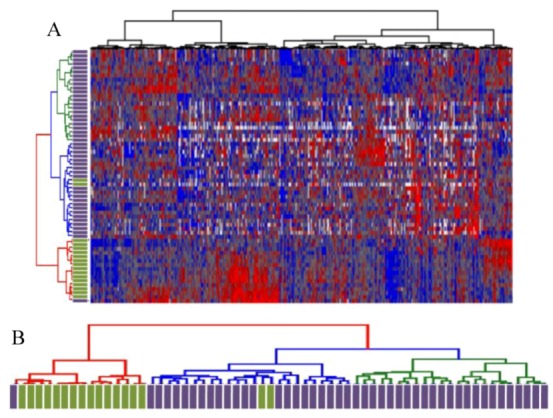
(**A**) Unsupervised hierarchical clustering of 596 highly variable genes. (**B**) Dendrogram depicting separation of MD-OS tumors (green bars) and LD-OS tumors based on expression of highly variable genes. (**A**) Unsupervised hierarchical clustering of OS tumor samples (Partek Genomics Suite) was performed on a subset of 596 genes found to be significantly differentially expressed within the dataset (expression varies by at least 6 fold, in at least 4 patients, *p* ≤ 0.001), according to methods previously described [13,14]. (**B**) The dendrogram reveals two clusters, one with red dendrogram lines that contains mostly metastatic-at-diagnosis tumors (green rectangles). A second cluster, which can be subdivided further (blue and green dendrogram lines) contains mostly localized-at-diagnosis tumors (purple rectangles).

Expression profiling was performed on 46 LD-OS tumor samples and 17 MD-OS tumor samples using UHN “human 19 k cDNA microarrays (H19K)”, that contain approximately 19,000 cDNA spots mapped to approximately 9,000 unique transcripts. Unsupervised gene discovery techniques were used to identify a subset of 596 genes that exhibited at-least six-fold change in expression in at-least four tumors (*p* ≤ 0.001), and hierarchical clustering of tumors according to the expression pattern of these genes demonstrated two broad groups of tumors. One group (Figure 1B, red dendrogram) contained all but two of the MD-OS tumors, and the other group (Figure 1B, green and blue dendrograms) contained all but one of the LD-OS tumors. This analysis supports the notion that osteosarcomas of varying metastatic status at the time of diagnosis exhibit distinct expression patterns, and that division of tumors into these categories (localized at diagnosis or metastatic at diagnosis) is appropriate for subsequent supervised analysis.

### 2.2. Differentially Activated and Organized Networks in Metastatic Osteosarcomas

**Figure 2:**
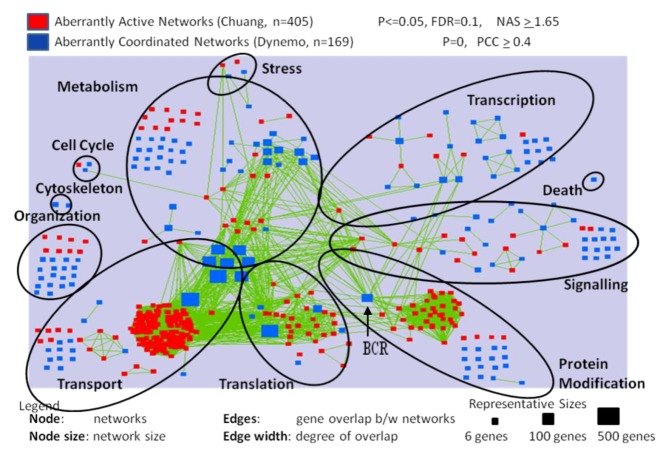
Nodes represent differentially activated (red squares) and organized (blue squares) networks in metastatic OS samples. Green edges represent genetic overlap between networks. Genes in significant networks were annotated with simplified Gene Ontology Slim Generic terms (Table 6), and networks were grouped into processes by the most commonly occurring term in each network. Networks meeting the cut-off conditions detailed at the top of the figure were visualized with the Enrichment Map plugin for Cytoscape. The size of each node reflects the number of genes included in the network.

The expression information was integrated with the Pathway Commons database of physical and genetic interactions [15], yielding a dataset of 5,855 genetic networks. Supervised analysis was performed on these data in a manner similar to previous publications to identify the differentially activated and organized networks in MD-OS expression profiles. To describe broad trends in human metastatic OS, inclusive cut-off levels were identified to delineate as many networks as possible that were significantly differentially activated or organized in the metastatic samples. For this analysis, four hundred and ninety seven (497) of the 5,855 genetic networks were identified as differentially activated and six hundred and eighty three (683) networks were significantly differentially organized. Networks annotated to transport, translation, organization and protein modification were more commonly differentially activated than expected by chance (Table 1, column 8). Networks annotated to the processes of organization, transport and translation were more commonly differentially organized than expected by chance, as were metabolic networks (Table 1, column 11). Three hundred thirty eight (338) differentially activated, and one hundred and sixty two (162) differentially organized, networks were visualized and clustered according to their Gene Ontology Process annotation (Figure 2). This visualization allowed the observation that clusters of differentially activated networks interact with large, significantly disorganized hubs (e.g., Figure 2—“protein modification” process: cluster of differentially activated networks interacting with the disorganized hub breakpoint cluster region (BCR). This pattern is also observed in the “translation” and “transport” processes.

**Table 1:** Enrichment of genetic networks in metastatic osteosarcomas according to cellular processes at the global (Permissive) significance level.

Cellular Processes	All Networks in Study				Significant Networks: Permissive Cutoffs					
	Subsets		Entire Networks		Differentially Activated			Differentially Organized		
1	2	3	4	5	6	7	8	9	10	11
	#	% of all	#	% of all	#	% of all	*p*-value	#	% of all	*p*-value
protein modification	573	9.8	575	9.8	52	10.5 *	5.3E-02	49	7.2	
transport	1002	17.1	869	14.8	194	39 *	1.1E-34 ^Ϯ^	133	19.5 *	9.0E-05 ^Ϯ^
signaling	954	16.3	955	16.3	69	13.9		87	12.7	
transcription	595	10.2	635	10.8	17	3.4		48	7	
stress	247	4.2	257	4.4	15	3		9	1.3	
metabolism	1340	22.9	1410	24.1	58	11.7		275	40.3 *	5.7E-24 ^Ϯ^
cell cycle	65	1.1	68	1.2	6	1.2 *	1.6E-01	7	1	
reproduction	130	2.2	137	2.3	6	1.2		0	0	
intracellular organization	195	3.3	201	3.4	27	5.4 *	3.4E-03 ^Ϯ^	54	7.9 *	9.1E-10 ^Ϯ^
development	338	5.8	357	6.1	0	0		0	0	
translation	146	2.5	99	1.7	50	10.1 *	4.4E-19 ^Ϯ^	17	2.5 *	3.9E-2 ^Ϯ^
death	31	0.5	36	0.6	0	0		3	0.4	
cytoskeleton	57	1	59	1	0	0		0	0	
ion transport	10	0.2	8	0.1	2	0.4 *	1.6E-01	0	0	
proliferation	3	0.1	2	0	1	0.2 *	2.1E-01	0	0	
homeostasis	2	0	2	0	0	0		0	0	
differentiation	6	0.1	6	0.1	0	0		0	0	
NaN	161	2.7	179	3.1	0	0		1	0.1	
**Totals**	**5855**	**100**	**5855**	**99.8**	**497**	**100**		**683**	**99.9**	

* denotes an enrichment (increase in proportion) above that found in the appropriate “all networks in study” category (e.g., column 7 is compared to column 3, and column 10 is compared to column 5). ^Ϯ^ denotes a significant *p*-value (*p* ≤ 0.05).

### 2.3. Genes Previously Implicated in Osteosarcoma Metastasis are among Significant Network Results

Next, it was examined if the genetic networks discovered to be differentially activated or organized in expression profiles of MD-OS samples contained genes previously implicated in OS metastasis. Extensive literature review led to the generation of a query list containing genes whose expression is either correlated with outcome in human OS patients, or genes that have been shown to be involved in OS metastasis in xenograft studies or animal models of OS (Table 2). Thirty-two genes from this list were included in the present study (*i.e.*, present on the expression profiling microarrays), and it was found that five such genes were among networks found to be significantly differentially activated in MD-OS expression profiles, and twenty-eight were among networks found to be significantly differentially organized in MD-OS expression profiles (Table 3 and Table 4). 

**Table 2:** Genes previously implicated in metastatic progression of OS.

Gene	Ref.	Gene	Ref.	Gene		Ref.
BIRC5	[16]	IVD	--	CXCR3	X	[17]
CAV1	[18]	KIT	[19]	EGF	X	[20,21]
CCN1	[22]	LRP5	[23]	EGFR2	X	[24,25,26,27,28,29]
CCN3	[30]	MMP2	[31]	FADD	X	[32]
CD44	[33]	PDGFRA	[34,35]	HIF1	X	[36]
CDH11	[37]	PEDF	[38]	IL12A	X	[39]
CDH2	[37]	RECK	[40]	IL12B	X	[39]
COL18A1	[41]	S100A6	[42]	INS	X	[43,44,45,46,47,48,49,50,51]
CXCR4	[52,53,54,55]	SPARC	[56]	MAML1	X	[57]
DPF2	--	TIMP1	[58]	MIRK	X	[59]
EGFR	[60]	PLAUR	[61]	MMP14	X	[62]
EZR	[25,63,64]	VEGFA	[55,65,66,67,68,69,70,71,72]	MMP9	X	[58,73,74]
FAS	[32,75,76,77]	VEGFB	[55,65,66,67,68,69,70,71,72]	PDGFA	X	[35]
IGF1	[43,44,45,46,47,48,49,50,51]	VEGFC	[55,65,66,67,68,69,70,71,72]	VCP	X	[78]
IGF2	[43,44,45,46,47,48,49,50,51]	WIF1	[79]			
IGF1R	[43,44,45,46,47,48,49,50,51]	YYI	[80]			

X denotes genes not present in the expression profiling microarrays used in this study.

**Table 3:** Differentially activated networks containing genes previously implicated in OS metastasis.

Query	Node	Score	Psample	FDRsample	Pgene	FDRgene
CDH2	CDH2	3.89	0	0.0017	0.037	0.019
S100A6	CACYBP	3.94	0	0.00096	0.007	0.18
TIMP1	ECH1	3.28	0	0.00096	0.003	0.17
PLAUR	PGAP1	2.54	0	0.0019	0.003	0.19
IVD	MECR	3.94	0.001	0.01	0.046	0.0076

“Node” is the central gene exhibiting differential network activity.

**Table 4:** Differentially organized networks containing genes previously implicated in OS metastasis.

Query	Node	ΔPCC_Total_	Psample	Query	Node	ΔPCC_Total_	Psample
S100A6	S100A6	0.41	0	TIMP1	LRP1	0.32	0
KIT	KIT	0.34	0	PLAUR	LRP1	0.32	0
YY1	YY1	0.32	0.001	BIRC5	PAFAH1B1	0.34	0
BIRC5	BIRC5	0.35	0.001	NOV	GIA1	0.3	0
MMP2	MMP2	0.35	0.001	VEGFA	SPARC	0.34	0
SPARC	SPARC	0.34	0	YY1	HDAC	0.33	0
IGF1R	CAMK2B	0.37	0	IGF1	PRKCD	0.36	0
IGF2	CAMK2B	0.37	0	PDGFRA	JAK1	0.33	0
EGFR	CAMK2B	0.37	0	CXCR4	JAK1	0.33	0
KIT	CAMK2B	0.37	0	FAS	BTK	0.35	0
CDH2	CAMK2B	0.37	0	VEGFB	RASA1	0.32	0
EZR	CAMK2B	0.37	0	MMP2	ITGB2	0.37	0
CD44	CAMK2B	0.37	0	LRP5	FZD8	0.48	0
SPARC	CAMK2B	0.37	0	SERPINF1	CSNK2A1	0.33	0.001
CYR61	ATP2A2	0.33	0	COL18A1	CTSL1	0.33	0.001
CAV1	ATP2A2	0.33	0	WIF1	FZD1	0.37	0.001
S100A6	ACTN1	0.32	0	IVD	MCCC1	0.37	0

“Node” is the central gene exhibiting differential network organization.

### 2.4. The PRKCε-RASGRP3-GNB2 Network Is Differentially Activated, and May Interact with the Disorganized DLG2 Hub

**Figure 3:**
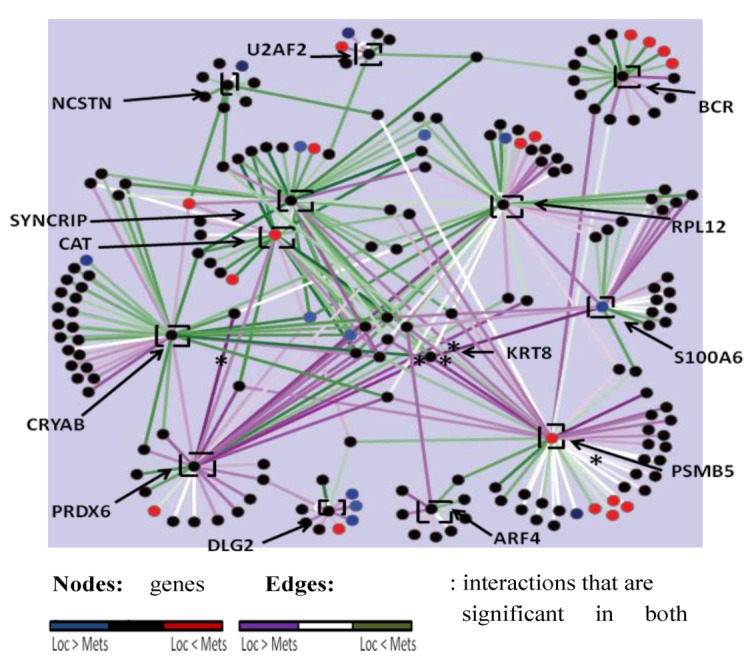
Labeled black squares show 12 differentially organized hubs (ΔPCC ≥ 0.4, *p* = 0, interactors ≥ 7). Nodes are genes and their colour reflects fold change in expression in the metastatic samples, only for significantly differentially expressed genes (t-test *p* ≤ 0.001). Only interactors of a significant hub whose expression is significantly correlated (PCC *p* ≤ 0.001) with expression of that hub in either the localized or metastatic samples are included. Edges depict interactions, and their colour reflects change in co-expression between metastatic samples and localized samples (green: correlation increased in metastatic samples, purple: correlation decreased in metastatic samples). Asterisks denote co-expressions that are significant (PCC *p* ≤ 0.001) in both localized and metastatic samples.

**Figure 4:**
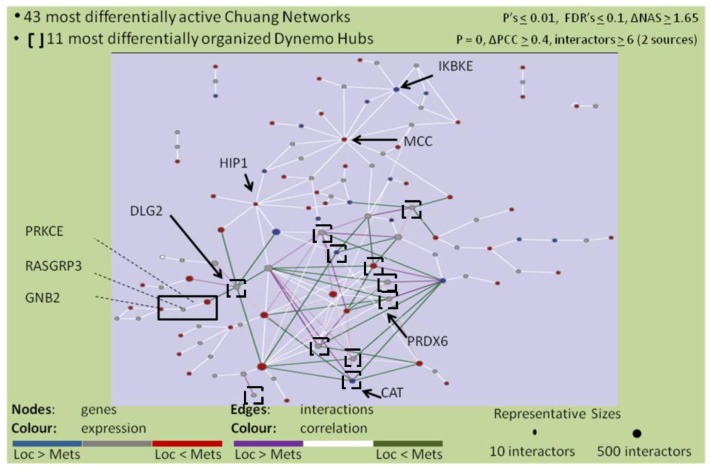
Genes comprising 43 differentially activated networks that meet stringent cut-offs (detailed at the top of the figure) were visualized with Cytoscape, as well as their interactions with 11 differentially organized networks (that also met stringent cut-offs which are detailed at the top of the figure). Coloured nodes represent relative abundance in metastatic samples *vs*. localized samples (red = more abundant, blue = less abundant). Coloured edges represent correlations between genes in metastatic samples *vs*. localized samples (green = more correlated, purple = less correlated).The number of included interactors of each gene in this study is proportional to the size of the node depicting the gene.

In order to identify drivers of the metastatic phenotype, the lists of significant networks discovered to be differentially activated and organized in expression profiles of MD-OS samples were further refined by focusing on a manageable number of each type of network that exhibited the greatest change between LD-OS and MD-OS samples. Twelve differentially organized networks were identified that exhibited the greatest change in organization between LD-OS and MD-OS samples, and that were also “genetic hubs” (having more interactors than the median), as it has been shown that these genes may be particularly important to cellular processes (Figure 3) [81]. Forty-three networks were identified that exhibited the greatest change in activity between LD-OS and MD-OS expression profiles, and their interactions with some of the most disorganized hubs were visualized (Figure 4). This analysis allowed the identification of the networks exhibiting the greatest change among MD-OS expression profiles, and also the observation that many differentially activated networks also interact with significantly disorganized hubs, e.g., the differentially activated network containing protein kinase C epsilon (PRKCε), RAS guanyl releasing protein 3 (RASGRP3), and guanine nucleotide binding protein 2 (GNB2), also interacts with the disorganized network of discs large homolog 2 (DLG2) (Figure 4). 

**Figure 5:**
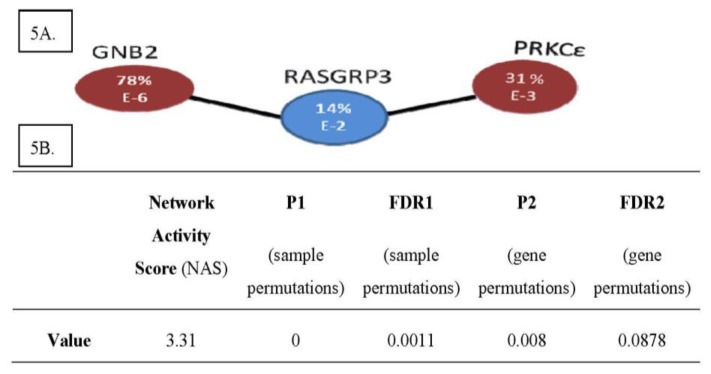
(**A**) The PRKCε-RASGRP3-GNB2 Network is Differentially Activated in Expression Profiles of Metastatic Osteosarcomas. (**B**) Differential Activity Score of PRKCε-RASGRP3-GNB2 Network. (**A**) Shown is the network of PRKCε-RASGRP3-GNB2 that is differentially activated in metastatic OS samples of the expression profiling screen. Red nodes are transcripts that are more abundant in metastatic samples, and blue nodes are less abundant in metastatic samples. Within each circle is the %-fold change in expression of metastatic samples *vs.* localized samples, and the order of magnitude of the *p*-value from the corresponding student’s t-test. (**B**) Shown are the relevant scores for the PRKCε-RASGRP3-GNB2 network, the results of the differential network activity analysis applied to expression profiles of MD-OS *vs.* LD-OS tumors.

Literature searches of the genes contained in the highly significant network results showed that PRKCε has been characterized in progression of prostate [82,83,84,85,86,87], breast [88,89], and renal cancers [90], as well as tumorigenesis of squamous cell carcinoma (non-melanoma skin [91,92,93,94,95], and head and neck [96,97,98,99]), as well as non-small cell lung cancer [100,101,102,103]. Additionally high expression of GNB2 is associated with an aggressive form of pulmonary adenocarcinoma (mixed adenocarcinoma with bronchioalveolar features), and shorter overall survival for patients with these tumors [104]. RASGRP3 has been shown to promote androgen independence and progression of prostate cancer [105]. These reports of the involvement of these genes in the progression of other cancers led to the selection of the differentially activated PRKCε-RASGRP3-GNB2 network as a lead candidate for further characterization in osteosarcoma cells and tumors (Figure 5).

### 2.5. PRKCε-RASGRP3-GNB2 Network Is Differentially Activated *in Vitro*

**Figure 6:**
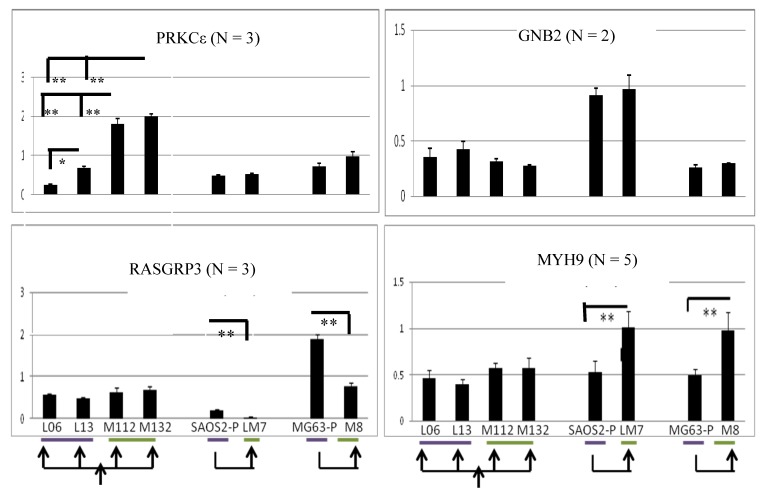
The PRKCε-RASGRP3-GNB2 network is differentially activated *in vitro.* Quantitative PCR was performed on cDNA synthesized from the human OS cell lines shown. Purple bars denote cell lines that are poorly metastatic in mouse models, and green bars denote cell lines that are strongly metastatic as murine xenografts. Arrows denote relationships between cell lines. Student’s t-test: * *p* ≤ 0.05 ** *p* ≤ 0.001. Multiple independent experiments are shown for each gene: GNB2 (n = 2), PRKCε and RASGRP3 (n = 3), and MYH9 (n = 5). ** *p* ≤ 0.001, * *p* ≤ 0.05.

**Figure 7:**
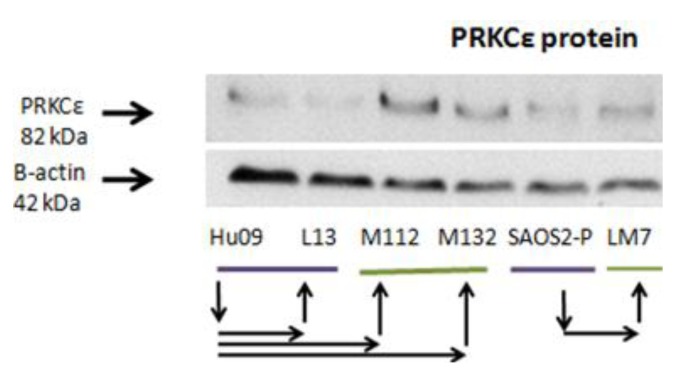
PRKCε Protein is more abundant in some highly metastatic cell line models of osteosarcoma metastasis. Western blots of whole cell lysates from human OS cell lines probed with anti-PRKCε and anti-β-actin antibodies. Purple bars denote cell lines that are poorly metastatic in mouse models, and green bars denote cell lines that are strongly metastatic in cell lines. Arrows denote relationships between cell lines. Shown is a representative western blot of two independent experiments.

A panel of human osteosarcoma cell lines known to have differing metastatic potential when grown as murine xenografts was collected to investigate the role of PRKCε-RASGRP3-GNB2. This panel includes the parental Hu-09 cell line (included in Figure 6), and its highly metastatic derivatives M112 and M132 (derived by *in vivo* metastatic selection), as well as the poorly metastatic sub-clones L06 and L13 (all generously provided by Dr. B. Fuchs, the University of Zurich, Zurich, Switzerland) [106,107]. Additionally the poorly metastatic SAOS2 and MG63 cell lines, as well as their highly metastatic derivatives LM7 and M8, were included (generously provided by Dr. E. Kleinerman, University of Texas MD Anderson Cancer Center, Houston, TX, USA) [108,109]. In this panel, it was observed that the PRKCε-RASGRP3-GNB2 network exhibited an mRNA expression pattern similar to that observed in the expression profiles of osteosarcoma tumors (Figure 6). Specifically, it was observed that PRKCε mRNA was significantly more abundant in some highly metastatic lines (M112 and M132 *vs.* L06 and L13), and that RASGRP3 was significantly less abundant in some highly metastatic lines (LM7 *vs.* SAOS2 and M8 *vs.* MG63). Additionally, mRNA levels of myosin chain heavy 9 (MYH9), which has been shown to interact with PRKCε at stress fibers in mice [110], was also observed to be more abundant in some highly metastatic lines (LM7*vs.* SAOS2 and M8*vs.* MG63). The protein expression of PRKCε was also investigated in this panel, and it was found that the highly metastatic lines M112 and M132 expressed PRKCε protein at higher levels than observed in either the less metastatic parental Hu09 line, or in the poorly metastatic L13 derivative (Figure 7).

### 2.6. Human Osteosarcomas That Are Metastatic-at-Diagnosis Are More Likely to Exhibit High Levels of PRKCε mRNA

**Figure 8:**
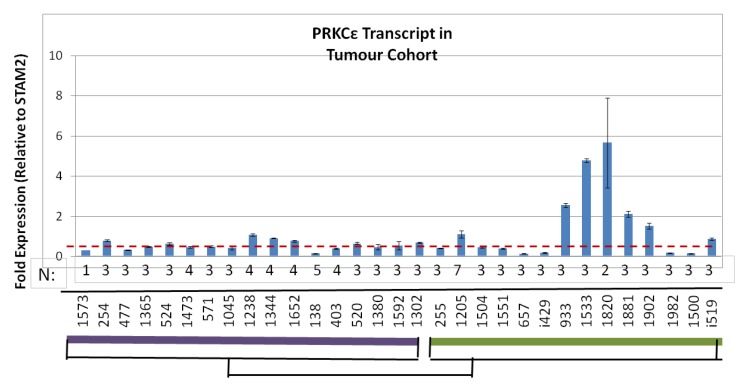
Metastatic-at-Diagnosis human osteosarcomas are more likely to have high PRKCε expression. Quantitative PCR was performed on cDNA synthesized from tumor samples from the original expression profiling cohort. PRKCε expression was normalized to STAM2, and is depicted as fold-change. The purple bar denotes localized tumors at diagnosis (LD-OS), and the green bar denotes metastatic tumors at diagnosis (MD-OS). The dotted red line depicts the average PRKCε expression in localized tumors. The number of replicates for each sample is shown below each bar.

The amount of PRKCε mRNA was assayed in 17 LD-OS and 14 MD-OS tumors that were used in the expression profiling screen (Figure 8). It was found that MD-OS tumors do not have a higher average expression of PRKCε than LD-OS tumors (Welch two sample t-test *p* = 0.1873), however MD-OS tumors were more likely to exhibit high expression of the PRKCε transcript (five of fourteen MD-OS tumors: Figure 8).

### 2.7. PRKCε Is Not Required for Migration of Highly Metastatic M132 Cells

**Figure 9:**
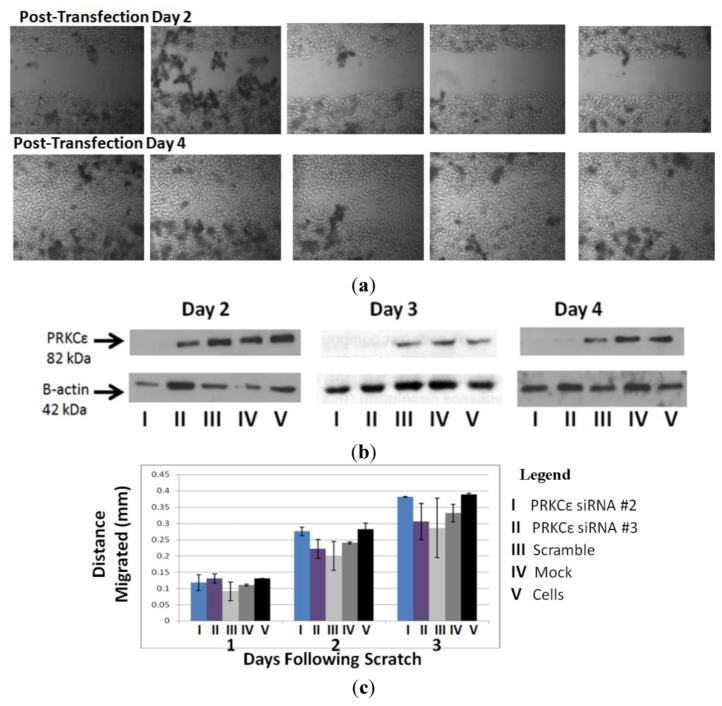
(**a**) PRKCε is not required for *in vitro* migration of highly metastatic M132 osteosarcoma cells. (**b**) Confirmation of PRKCε knockdown. (**c**) Quantification of migration by M132 cells. (**a**) Upper panel: photographs of M132 cells immediately after scratching, two days following transfection with PRKCε siRNA. Lower panel: matched photographs of M132 cells two days following scratching and four days following transfection with PRKCε siRNA. (**b**) Western blot verification of PRKCε knock-down two, three and four days following transfection with siRNA. (**c**) Quantification of migrated distance by M132 cells transfected with PRKCε siRNA and appropriate controls. Values are average ± standard error of three independent experiments.

Since PRKCε promotes the *in vitro* migration of other cell lines [89], and the *in vitro* migration rate of the Hu09-derived cell lines correlates with their ability to form metastatic colonies in mice [107], experiments were undertaken to determine whether PRKCε promotes migration of osteosarcoma cells. The highly metastatic M132 cell line was selected for PRKCε-knockdown studies, as this line expressed PRKCε protein at a high level (Figure 7). However, it was observed that knockdown of PRKCε protein using siRNA did not affect the *in vitro* migration rates of M132 cells as observed during a scratch assay (Figure 9).

### 2.8. IGF-1 Stimulation Induces Protein Expression of PRKCε in M112 Osteosarcoma Cells

It is well known that the insulin/insulin-like growth factor (IGF) pathway plays an important role in osteosarcoma tumor growth. Osteosarcoma tumors and cell lines express components of the pathway (including insulin, IGF-I and -II, as well as pathway receptors) that are capable of autocrine signaling [44,45,51]. Additionally, inhibition of the pathway through various means is effective at inhibiting osteosarcoma growth in xenograft models [43,46,47,48,49,50]. IGF-I signaling is also known to lead to increased cellular levels of diacylglycerol, which can then activate PRKCε (and other proteins containing C1 domains) [111,112,113], by a mechanism involving membrane tethering of PRKCε and conformational change [114]. Specifically PRKCε is activated following IGF-1 treatment in vascular smooth muscle cells, and may be involved in IGF-I-mediated proliferation and migration of these cells [112,113]. 

**Figure 10:**
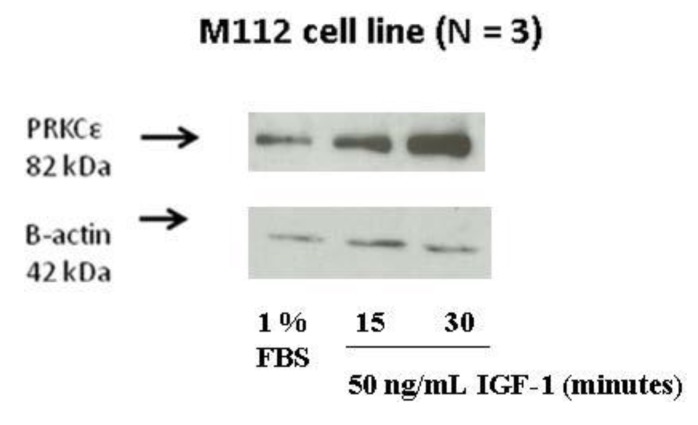
Protein expression of PRKCε is induced by IGF-1 treatment. Western blots were performed on whole cell lysates of M112 cells incubated in 1% FBS (low serum) medium or in 1% FBS medium supplemented with 50 ng/mL IGF-1 for the time periods described. Membranes were probed with anti-PRKCε antibody and anti-β-actin antibody as a loading control. The experiment was performed three independent times.

The effect of IGF-1 stimulation on PRKCε protein in highly metastatic osteosarcoma cells was investigated. Highly metastatic M112 osteosarcoma cells were serum starved for at least 24 h, followed by the addition of either fresh serum-free media or media containing 50 ng/mL IGF-1. It was observed that following IGF-1 stimulation, the protein expression of PRKCε increased in a time-dependent manner, with a peak occurring approximately 30 min following stimulation (Figure 10).

## 3. Discussion

This work demonstrates that expression profiles of metastatic-at-diagnosis osteosarcomas (MD-OS) are quite distinct from expression profiles of localized-at-diagnosis osteosarcomas (LD-OS), possibly indicating a distinct disease etiology for the more severe MD-OS. Supervised network analysis discovered hundreds of networks that exhibited both differential activity and differential organization in the MD-OS expression profiles, at permissive cut-off levels. This indicates that either heterogeneous differences are observed in the MD-OS tumor group, or that changes to the transcriptome observed in MD-OS are potentially associated with many bystander events (*i.e.*, aberrations in expression pattern not functionally relevant to osteosarcoma metastasis), or possibly both.

Investigation of the cellular processes of networks within the permissive significant results showed differentially activated networks were strongly enriched for transport and translation networks, and slightly enriched for intracellular organization networks. Differentially organized networks were strongly enriched for metabolic networks, as well as intracellular organization and transport networks. Increased or over-active translation is known to support an aggressive phenotype of many cancers [115], and the analysis presented here implies it is a characteristic of MD-OS tumors as well. The enrichment of disorganized metabolic networks in MD-OS expression profiles may indicate that altered cellular metabolism plays a role in osteosarcoma metastasis, a notion supported by a recent study by Hua *et al*. [116]. By studying serum metabolite profiles of mice injected with OS cells, Hua et al observed a metabolic shift coincident with the onset of pulmonary metastasis [116].

Examination of the most stringently differentially activated and organized networks in MD-OS led to the selection of the PRKCε-RASGRP3-GNB2 network for follow-up characterization. This network was subsequently found to be differentially activated at the transcript level among a panel of *in vitro* models of OS metastasis. PRKCε was also found to be more abundant in some highly metastatic OS cells at the protein level, and in some MD-OS tumors at the transcript level. Although PRKCε is known to support migration of other cell types [89,96,98,117,118], there was no evidence to support a role for PRKCε in the migration of M132 osteosarcoma cells in this study. This may indicate that the aberrant expression of PRKCε and its network *in vitro* and *in vivo* is related to a bystander effect, or that the functional relevance of this aberrant expression has not yet been elucidated. The effect of high PRKCε expression may be related to the proliferation effects of the IGF-1 pathway, as this study provides evidence for the IGF-1 dependent induction of PRKCε protein expression. PRKCε may support other cellular processes entirely, as it is known to promote pro-metastatic phenotypes of many other cancers [82,83,84,85,86,87,91,92,93,94,95,96,97,98,99,100,101,102,103].

In addition to the PRKCε-RASGRP3-GNB2 and MYH9 network; this article describes several networks exhibiting significant differential activity, organization, or some combination of the two in MD-OS expression profiles. The integration of the results of this study with other datasets of osteosarcoma expression profiles, as they become available, will help to distinguish the drivers and genuine characteristics of metastatic osteosarcoma from the passengers, as would a limited high-throughput functional screen of the significant networks described in this study.

## 4. Experimental Section

### 4.1. Patient Follow-Up

Overall survival data was available for all 46 patients in the group presenting without metastasis at diagnosis. Of these, 17 died of disease (DOD) with a median follow-up of 41 months (minimum follow-up = 6 month, maximum follow-up = 157, SD = 39.2 months), 28 are alive with no evidence of disease (ANED), with a median follow-up of 101 months (minimum follow-up = 35 month, maximum follow-up = 269, SD = 62.1 months) and one subject is alive with evidence of disease (AWED) with a follow-up of 12 months.

Out of 17 patients presenting with metastases, overall survival data was available for only 14 patients. Out these 14, 13 died of disease (DOD) with a median follow-up of 10 months (minimum follow-up = 1 month, maximum follow-up = 49, SD = 12.5 months) and the other patient is alive with no evidence of disease (ANED) with a follow-up of 178 months.

### 4.2. Tumor Samples

Primary high-grade intramedullary osteosarcoma tumors were selected for expression profiling by sarcoma pathologists on the basis of tumor homogeneity. The OS tumor cohort consisted of 63 tumors which were grouped into those that were localized (n = 46) or metastatic (n = 17) at the time of initial diagnosis. Samples were collected by open biopsies prior to administration of any chemotherapy, and stored in liquid nitrogen until time of RNA isolation. Total RNA was extracted using Trizol reagent (Invitrogen, Carlsbad, CA, USA). The amount and quality of RNA was assessed using both Ultrospec 2100 *pro* (GE Healthcare Bio-Sciences, Piscataway, NJ, USA) and 1% agarose gels.

### 4.3. Gene Expression Profiling

5 μg of tumor and “reference” (pooled from cell lines) cDNA was indirectly labeled using aminoallyl nucleotide analogues with Cy3 and Cy5 fluorescent tags, respectively. The labeled cDNA was competitively hybridized to University Health Network 19 k cDNA arrays (UHN19k) containing 18,981 “spots” (mapped to 8,998 known unique genes). This process was repeated with reciprocal fluorescent tagging. Data normalization, imputation (K10 Nearest Neighbours algorithm), and analysis were performed in collaboration with Drs. Shelley Bull, Dushanthi Pinnaduwage, and Robert Parkes. Supervised statistical analysis (random variance T-test) was performed by Robert Parkes using BRB-Array Tools software [119]. 

### 4.4. Unsupervised Hierarchical Clustering

The most differentially expressed single genes within the expression profiling experiment were identified according to methods previously described [13,14]. In this study, a subset was examined that exhibited at least six-fold change in expression in at-least four tumors with a maximum *p*-value of 0.001 (student’s t-test). Unsupervised hierarchical clustering of the tumors according to their expression of these genes was performed with Partek Genomics Suite.

### 4.5. Supervised Network Analysis

The entire database of interactions for human genes and proteins was downloaded from the Pathway Commons website (as an adjacency list and the interactions were converted to Entrez GeneIDs [15]. This dataset comprised physical and genetic interactions, as well as pathway and disease associations (which were either translated by Pathway Commons to binary interactions or were lost), from both curated and non-curated sources [15]. A subset of 5,855 genes was common to both this interaction database and the expression profiling experiment. There were 176,121 interactions among these genes, with six interactions being the median number per gene.

#### 4.5.1. Differentially Activated Networks

Genetic networks demonstrating significant differential “activity” in MD-OS tumors were discovered in a manner similar, with some changes, to that previously described by Chuang *et al.* [11]. Briefly each network was restricted to those genes that met some cut-off of significant expression between localized and metastatic tumors (Table 5). 

**Table 5:** Cut-Off conditions for gene inclusion to “differential activity” analysis.

Trial	1	2	3	4	5	6	7	8	9	10
Differential Expression (%)	0	0	10	20	30	40	50	60	70	80
P Value Maximum	None (*i.e.*, all genes included)	0.001								

For each gene in the remaining network a “class difference score” (CDS) was calculated as the difference in median expression between classes (*i.e.*, between metastatic tumors and localized tumors), and normalized to the variation within the localized samples [Equation (1)]. A “network activity score” (NAS) was calculated by determining the average of the absolute CDS for each gene in the network [Equation (2)]. In these equations, g_1_, g_2_… gn are all genes in network J, which has N_G_ members.G_A_ and G_B _are the median expression of gene G among localized (A) and metastatic (B) tumors. S_GA_ is the standard deviation of expression values of gene G among the localized tumors:

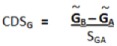
(1)

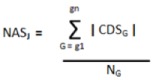
(2)


To determine statistical significance, the NAS was compared to the corresponding NAS generated from 1,000 permutations of both sample and gene labels to determine two empirical *p*-values [Equation (3)]. In these equations J1 … ɸ − 1, ɸ, ɸ + 1 … Ĵ are all networks in the study, and i … ɫ - 1, ɫ, ɫ + 1 … N_i_ are the repetitions of gene and label permutations (in this study N_i_ = 1,000).



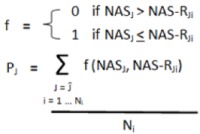
(3)


The significance of the randomly generated NAS scores was also determined in a similar fashion [Equation (4)]; this was done for false-discovery rate (FDR) calculation. 


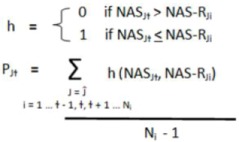
(4)

Two FDRs were calculated for each network by determining the average number of randomly-generated networks with a *p* value equal to or lower than a nominal *p* value threshold [*p*_ɸ_ in Equation (5) below], equal to the *p*-value of the network being considered. The number of randomly generated *p*-values falling below this nominal threshold was then divided by the number of real networks also falling below this nominal threshold [Equation (5)]. This was done for both *p*-values (from sample and gene permutations) to yield two empirical FDR values for each network. The entire process was repeated for different cut-off conditions (Table 5), and was stopped when more stringent cutoffs failed to produce any additional significant networks:



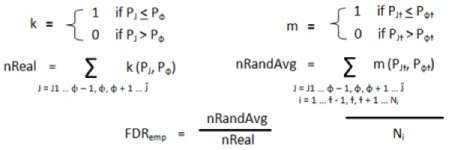
(5)


#### 4.5.2. Differentially Organized Network—Dynemo

Genetic networks exhibiting significant differential organization in MD-OS samples relative to localized samples were discovered as previously described [12]. Briefly, for each network the Pearson Correlation Coefficient (PCC) was used to determine the overall correlation in gene expression between the network hub and each of its interactors in both the localized and metastatic samples [Equation (6)]. The difference in this hub-interactor correlation between localized and metastatic samples was then calculated for each interaction in the network [Equation (7)], and the average difference in correlation across the entire network was also calculated: “AvgΔPCC” [Equation (8)]. In these equations J1 … ɸ − 1, ɸ, ɸ + 1 … Ĵ are all the networks in the study. H is the hub (central node) of network J, which has N_G_ members, and g1, g2 … gn are all interactors of H, and therefore all other genes in network J. S_G_ and S_H_ are the standard deviations of gene G and hub H among the indicated tumor class. t1, t2 … T are all the tumors in each class, and N_T_ is the total number of tumors in the localized (N_T_ = 46) and metastatic (N_T_ = 17) classes:

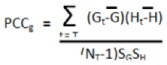
(6)

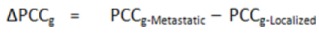
(7)

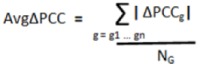
(8)


The AvgΔPCC value was compared to the corresponding scores generated from the same network following 1,000 permutations of the class labels to generate a non-parametric *p*-value to assess the significance of the change in internal correlation of each network [Equation (9)]. Let i … ɫ − 1, ɫ, ɫ + 1 … N_i_ be the number of repetitions of gene and label permutations (in this study N_i_ = 1,000).


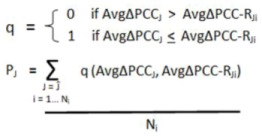
(9)

#### 4.5.3. Visualization of Network Results

Two statistical confidence levels were investigated in this study, a permissive cut-off level to discover broad (or “global”) trends among metastatic tumors, and a stringent cut-off to delineate high-confidence networks for follow-up. The permissive cut-off for differentially activated networks was chosen to be *p*’s ≤ 0.05 and FDR’s ≤ 0.2, and for the differentially organized levels the permissive cut-off was *p* ≤ 0.001. These cut-offs were used to assess the significance of cellular process enrichment at the global level (Table 1) and for discovery of networks containing genes previously implicated in OS metastasis (Table 3 and Table 4). Visualization of these global trends (Figure 1) was limited to computer processing power, and thus were further refined for the differentially activated networks to be *p*’s = 0.05, FDR’s = 0.1 and NAS ≥ 1.65, and for differentially organized networks to be *p* ≤ 0.001 and |ΔPCC| ≥ 0.4. Visualization of the global trends was performed with the Enrichment Map plugin for Cytoscape [120]. The stringent cut-off levels for differentially activated networks were set to *p*’s ≤ 0.01, FDR’s ≤ 0.1, NAS ≥ 1.65, and for differentially organized networks were set to *p* = 0, |ΔPCC| ≥ 0.4, and having at least seven interactors. Visualization of these high-confidence networks was conducted using Cytoscape [121] (Figure 3 and Figure 4).

#### 4.5.4. Cellular Process Annotation

**Figure 11:**
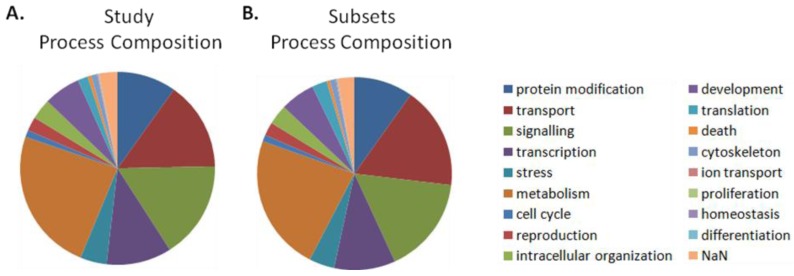
Distribution of cellular processes among networks and sub-networks investigated in this study. (**a**) “Study” Process Composition (**b**) “Subsets” Process Composition. (**a**) The Generic Slim database of gene-process associations was accessed from Gene Ontology and each network was annotated according to the most commonly occurring process term among genes within the network. (**b**) For all 497 networks found to be differentially activated in this study, the networks were re-annotated according to the most commonly occurring term among the significant sub-set (as the analysis of differential activity involves identification of a significant subset of genes within each network).

Gene Ontology annotations (which relate genes to cellular processes) from the “Generic Slim” database were downloaded [122]. The database was further simplified to focus on interesting processes according to Table 6. Networks were assigned to processes by determining the most commonly occurring term among genes within the network. In this manner all 5,855 networks in the study could be assigned to a process (Figure 11a—the “study process composition”, or Table 1, column 4). Since analysis of “differential activated” networks requires identification of significant subsets, all networks which were eventually found to be significantly differentially activated were re-annotated using only the genes within the network’s significant subset (Table 1, column 2). This resulted in discordant annotations between the network and the significant subset for only 6% of all networks, and thus the annotations of the network subsets (Figure 11b—“subsets process composition”, or Table 1, column 2) and overall “study process composition” (Figure 11a, or Table 1, column 4) are overwhelmingly similar. As the methods of Taylor *et al.* do not identify significant subsets within networks, this consideration was not necessary for differentially organized networks.

**Table 6:** Simplification of the gene ontology slim generic database.

Original Terms	Further Simplified Terms
cell death, death	death
multicellular organismal development, embryonic development, anatomical structure morphogenesis	development
cell differentiation, differentiation	differentiation
regulation of gene expression, epigenetic	epigenetics
cell growth, growth	growth
cellular component organization, organelle organization, mitochondrion organization, cytoplasm organization	intracellular organization
metabolic process, cellular amino acid and derivative metabolic process, secondary metabolic process, lipid metabolic process, biosynthetic process, catabolic process, carbohydrate metabolic process, protein metabolic process, nucleobase nucleoside nucleotide and nucleic acid metabolic process, DNA metabolic process, generation of precursor metabolites and energy	metabolism
signal transduction, response to biotic stimulus, response to external stimulus, response to abiotic stimulus, cell-cell signaling, cell communication, response to endogenous stimulus, cell recognition	signaling
protein transport, transport	transport
regulation of biological process, biological process, behavior	NaN

#### 4.5.5. Cellular Process Enrichment

Enriched cellular processes were determined separately for differentially activated and organized networks. Enriched processes were first identified by determining those processing comprising a greater proportion within the significant results than in the entire study (*i.e.*, differentially activated: Table 1 column 7 was compared to Table 1 column 3, differentially organized: Table 1 column 10 was compared to Table 1 column 5). 

Significance of cellular process enrichment (*p*-value) within the network results was determined using the hypergeometric distribution [Equation (10)], as described by Boyle *et al*. [123]. In this equation, N is the total number of networks in the study (5,855). M is the number of networks in the entire study annotated to a process of interest (differentially activated: Table 1 column 2, differentially organized: Table 1 column 4). *i* is the number of networks within the significant results annotated to the same process of interest (differentially activated: Table 1 column 6, differentially organized: Table 1, column 9), and n is the number of networks contained within that set of significant results (bottom of Table 1—differentially activated: 497, differentially organized: 683). This operation was performed with Matlab using the “hygepdf” command.



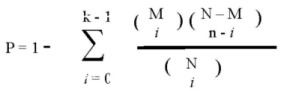
(10)


### 4.6. Cell Culture

Hu09 and derivates (L06, L13, M112 and M132) were grown in RPMI 1640 supplemented with 10% fetal bovine serum (FBS) and 1% L-glutamine. L06 and L13 are cell lines derived from Hu09 human OS cell line by limited dilution plating, and M112 and M132 were derived from the Hu09 line by *in vivo* selection of pulmonary metastatic nodules [106,107]. L06 and L13 both form fewer lung metastases at a decreased incidence in mice following tail-vein injection compared to the intermediate Hu09 line, while M112 and M132 result in greater numbers of pulmonary nodules at a higher incidence [107]. The differences in metastatic propensity correlate to different survival lengths for mice injected with the various cell lines [107]. M8 cells were derived from MG63 human OS cells (hereafter referred to as MG63-P), and have decreased latency until pulmonary metastasis [109]. Both were grown in Dulbecco’s Modified Eagle Medium supplemented with 10% FBS. LM7 cells were derived from SAOS2 human OS cells (hereafter referred to as SAOS2-P), and form pulmonary metastases at a greater incidence than SAOS2-P following tail-vein injection in mice [108]. LM7 and SAOS2-P were grown in McCoy’s 5A supplemented with 5% FBS. RNA was harvested from all cell lines using Trizol reagent followed by phenol/chloroform extraction.

### 4.7. Quantitative Reverse-Transcription Polymerase Chain Reaction (rt-PCR)

RNA was collected from OS tumor samples as previously described [124], and cDNA was synthesized from both tumor RNA and cell line RNA according to the manufacturer’s instructions (M-MLV Reverse Transcriptase—Invitrogen, Carlsbad, CA, USA). Quantitative rt-PCR was performed according to the manufacturer’s instructions (Sybr Green—Applied Biosystems, Life Technologies, Carlsbad, CA, USA) to quantify the abundance of target cDNA relative to that of a control gene, signal transducing adaptor molecule 2 (STAM2), using primer sequences according to Table 7. Dr. Dushanthi Pinnaduwage performed statistical analysis (Welch two-sample t-test on log2 transformed data) comparing expression levels of protein kinase C epsilon (PRKCε) transcript between localized and metastatic tumors.

**Table 7:** Primer sequences used in this study.

Gene Symbol	Primer Pairs
STAM2	Forward 5'-TGGATGACAGTGATGCCAATTG-3'
	Reverse 5'-CGCTGCCTCAGTCTCTATGT-3'
PRKCε	Forward 5'-CACTGCAAGCTGGCTGACT-3'
	Reverse 5'-TGCAGGATCTCAGGAGCTATG-3'
RASGRP3	Forward 5'-GGATTTCTCTGGGGCATAATC-3'
	Reverse 5'-AGGAGGTCTTTGCACTGTTTG-3'
GNB2	Forward 5'-CTATCAAGCTGTGGGACGTG-3'
	Reverse 5'-GTAGCCGTTGGGGAAGAAAG-3'
MYH9	Forward 5'-GCCTACAGGAGTATGATGCAAG-3'
	Reverse 5'-ACTGGATGACCTTCTTGGTGTT-3'

### 4.8. Western Blots

Cytosolic protein extracts were isolated from cell lines using NETN lysis buffer (150 mM NaCl, 1 mM EDTA, 20 mM Tris pH 7.5, 0.5% NP40, 1 mM phenylmethylsulphonyl fluoride, and 1% each of protease inhibitor, phosphatase inhibitor I and phosphatase inhibitor II, all from Sigma-Aldrich, (St. Louis, MO, USA). Protein concentration was determined using the bicinchoninic acid (BCA) protein assay kit (Pierce, Thermo Scientific, Rockford, IL, USA). Proteins were separated using 10% sodium dodecyl sulfate polyacrylamide gel electrophoresis (SDS-PAGE) and transferred to nitrocellulose membranes at 30 V, overnight at 4 °C. Membranes were blocked for 1 h with Tris buffered saline with 0.1% Tween-20 (TBS-T) supplemented with 5% fat-free milk. Primary antibody incubation was performed in TBS-T supplemented with 5% bovine serum albumin (BSA) or fat-free milk, according to the manufacturer’s instructions. Secondary antibody incubation (donkey anti mouse, donkey anti rabbit) was performed at 1:5,000 concentration in TBS-T with 5% BSA for 50 min. Protein bands were visualized by chemiluminescence using ECL detection system (Amersham, GE Healthcare Bio-Sciences Corp, Piscataway, NJ, USA,). Primary antibodies used in this study are PKCε (1:1,000, Cell Signaling Technology, Danvers, MA, USA), and β-actin (1:5,000, Sigma-Aldrich, St. Louis, MO, USA). Analysis of PRKCε protein expression in the panel of OS cell lines was performed in triplicate.

### 4.9. Knockdown of PRKCε

Two different siRNAs were purchased from Ambion (Invitrogen, Carlsbad, CA, USA) targeting PRKCε: “select s11102” and “select s11103” (hereafter referred to as PRKCε siRNA #2 and #3, respectively). Ambion “select negative control siRNA #1” was used as a negative control (hereafter referred to as “scramble”). Transfection reagents were purchased from Dharmacon Thermo Scientific (Rockford, IL, USA): “DharmaFECT 2” was used for the M112 cell line, and “DharmaFECT 4” was used for the M132 cell line. Transfections were performed in parallel with cell plating for experiments. 20 μM siRNA and the appropriate transfection reagent were each diluted (1.5:100 and 1:100, respectively) in Opti-MEM medium (Invitrogen, Carlsbad, CA, USA) and left to incubate for 5 min at room temperature (RT). The siRNA and transfection reagents were then mixed together and incubated for 10 min at RT. 200 μL of the mixture was then applied to wells of 12-well plates, or 20 μL was applied to wells of 96-well plates. 800 μL (12-well plates) or 80 μL (96-well plates) of cells at an appropriate concentration were then plated evenly in the wells. The cells were washed with phosphate-buffered saline (PBS) the following day and given fresh media.

### 4.10. Scratch Assay

Comparison of migration rate following knockdown of PRKCε in M132 cells was performed by plating 1.2 × 10^5^ cells in 12-well plates. All plates had grids drawn across them to allow repeated photographing of the same field. At least 12 fields were collected and analyzed for each sample on each day. Twenty four hours following plating, the cells were washed with PBS and given RPMI 1640 supplemented with 1% each of FBS and L-glutamine (“low serum media”). On the second day following plating, the confluent cells were “scratched” using a 200 μL pipette tip. The cells were washed twice with PBS and fresh low serum media was added before immediately imaging the cells using an inverted microscope and camera. The average distance that the confluent edge of cells had travelled into the wound was measured for each time point. This experiment was performed in triplicate.

### 4.11. IGF-1 Induction Assay

To determine if IGF-1 is able to induce the expression of PRKCε, 2 × 10^5^ M112 or M132 cells were plated in 12-well plates. After two days the cells were washed twice with PBS and were given low serum RPMI media (1% FBS, 1% L-glutamine), with the exception of some cells which were retained in complete media as a control. The following day the remaining cells were given fresh low serum RPMI supplemented either with nothing (“no treatment” control) or with 50 ng/mL of IGF-1 (Sigma-Aldrich, St. Louis, MO, USA). After the appropriate length of incubation with IGF-1, the cells (4 wells) were washed with cold PBS, scraped, and then lysed in order to harvest cytosolic protein as described above. Trizol reagent was added to a fifth well of each sample for the extraction of RNA and evaluation of mRNA abundance as described above. This experiment was repeated in triplicate.

## 5. Conclusions

Supervised network analysis was used to discover differentially activated and organized genetic networks in expression profiles of metastatic-at-diagnosis osteosarcomas (MD-OS) compared to localized-at-diagnosis osteosarcomas (LD-OS). The PRKCε-RASGRP3-GNB2 network was found to be differentially activated among MD-OS expression profiles and among *in vitro* models of OS metastasis. It was found that MD-OS tumors do not express significantly higher levels of PRKCε overall (t-test *p* = 0.1873), but they were more likely to exhibit high expression of PRKCε transcript, compared to the LD-OS tumors (five of fourteen MD-OS tumors had PRKCε expression greater than the maximum of the LD-OS tumors).

This result is consistent with the expression pattern observed in the panel of OS cell lines, where PRKCε was found to be more abundant at the RNA level in some of the *in vitro* models of OS metastasis. Specifically, PRKCε was more abundant in the M112 and M132 *vs*. Hu09, L06 and L13 cell line model, but not in the LM7 *vs*. SAOS2 or M8 *vs*. MG63 models. The heterogeneity of PRKCε expression among MD-OS tumors may indicate heterogeneous networks are aberrant in MD-OS tumors, or that the PRKCε-RASGRP3-GNB2 network may be disrupted through alterations of other genes in the network.

Despite reports by others of the involvement of PRKCε in cell migration [89,90,96,98,118], knockdown of PRKCε using siRNA was not found to affect migration of highly metastatic M132 osteosarcoma cells. The effect of PRKCε on invasion of osteosarcoma cells was not investigated in this study, and may be a fruitful avenue of further research, as PRKCε is known to be involved in invasion of other cell systems *in vitro*, as well as to be involved in various other pro-metastatic pathways [125,126]. The absence of pro-metastatic effect of PRKCε protein in migration assays indicates that either the aberrant expression of the PRKCε-RASGRP3-GNB2 network may be related to a bystander effect, or that the pro-metastatic phenotype of PRKCε over-expression remains to be elucidated.

PRKCε protein expression in highly metastatic M112 cells was found to be induced by IGF-1 stimulation, and this may indicate that PRKCε is involved in IGF-1 dependent pathways. Beyond the PRKCε-RASGRP3-GNB2network, this article describes many aberrantly activated and organized networks among expression profiles of MD-OS tumors. A systematic functional screen of these networks, and determination of the predictive accuracy of these network expression patterns in independent datasets, would help differentiate the bystanders from the drivers of OS metastasis. 

## References

[1] Kansara M., Thomas D.M. (2007). Molecular pathogenesis of osteosarcoma. DNA Cell Biol..

[2] Murphey M.D., Robbin M.R., McRae G.A., Flemming D.J., Temple H.T., Kransdorf M.J. (1997). The many faces of osteosarcoma. Radiographics.

[3] PosthumaDeBoer J., Witlox M.A., Kaspers G.J., van Royen B.J. (2011). Molecular alterations as target for therapy in metastatic osteosarcoma: A review of literature. Clin. Exp. Metastasis.

[4] Clark J.C., Dass C.R., Choong P.F. (2008). A review of clinical and molecular prognostic factors in osteosarcoma. J. Cancer Res. Clin. Oncol..

[5] Kong C., Hansen M.F. (2009). Biomarkers in Osteosarcoma. Expert Opin. Med. Diagn..

[6] Schulze A., Downward J. (2001). Navigating gene expression using microarrays—A technology review. Nat. Cell Biol..

[7] Dupuy A., Simon R.M. (2007). Critical review of published microarray studies for cancer outcome and guidelines on statistical analysis and reporting. J. Natl. Cancer Inst..

[8] Goeman J.J., Buhlmann P. (2007). Analyzing gene expression data in terms of gene sets: Methodological issues. Bioinformatics.

[9] Song S., Black M.A. (2008). Microarray-based gene set analysis: A comparison of current methods. BMC Bioinformatics.

[10] Subramanian A., Kuehn H., Gould J., Tamayo P., Mesirov J.P. (2007). GSEA-P: A desktop application for Gene Set Enrichment Analysis. Bioinformatics.

[11] Chuang H.Y., Lee E., Liu Y.T., Lee D., Ideker T. (2007). Network-based classification of breast cancer metastasis. Mol. Syst. Biol..

[12] Taylor I.W., Linding R., Warde-Farley D., Liu Y., Pesquita C., Faria D., Bull S., Pawson T., Morris Q., Wrana J.L. (2009). Dynamic modularity in protein interaction networks predicts breast cancer outcome. Nat. Biotechnol..

[13] Perou C.M., Sorlie T., Eisen M.B., van de Rijn M., Jeffrey S.S., Rees C.A., Pollack J.R., Ross D.T., Johnsen H., Akslen L.A. (2000). Molecular portraits of human breast tumours. Nature.

[14] Van’t Veer L.J., Dai H., van de Vijver M.J., He Y.D., Hart A.A., Mao M., Peterse H.L., van der Kooy K., Marton M.J., Witteveen A.T. (2002). Gene expression profiling predicts clinical outcome of breast cancer. Nature.

[15] Cerami E.G., Gross B.E., Demir E., Rodchenkov I., Babur O., Anwar N., Schultz N., Bader G.D., Sander C. (2011). Pathway Commons, a web resource for biological pathway data. Nucleic Acids Res..

[16] Osaka E., Suzuki T., Osaka S., Yoshida Y., Sugita H., Asami S., Tabata K., Hemmi A., Sugitani M., Nemoto N. (2006). Survivin as a prognostic factor for osteosarcoma patients. Acta Histochem. Cytochem..

[17] Pradelli E., Karimdjee-Soilihi B., Michiels J.F., Ricci J.E., Millet M.A., Vandenbos F., Sullivan T.J., Collins T.L., Johnson M.G., Medina J.C. (2009). Antagonism of chemokine receptor CXCR3 inhibits osteosarcoma metastasis to lungs. Int. J. Cancer.

[18] Cantiani L., Manara M.C., Zucchini C., de Sanctis P., Zuntini M., Valvassori L., Serra M., Olivero M., di Renzo M.F., Colombo M.P. (2007). Caveolin-1 reduces osteosarcoma metastases by inhibiting c-Src activity and met signaling. Cancer Res..

[19] Entz-Werle N., Marcellin L., Gaub M.P., Guerin E., Schneider A., Berard-Marec P., Kalifa C., Brugiere L., Pacquement H., Schmitt C. (2005). Prognostic significance of allelic imbalance at the c-kit gene locus and c-kit overexpression by immunohistochemistry in pediatric osteosarcomas. J. Clin. Oncol..

[20] Boulytcheva I.V., Soloviev Y.N., Kushlinskii N.E., Mahson A.N. (2010). Expression of molecular markers in the tumor and survival prognosis in osteosarcoma. Bull. Exp. Biol. Med..

[21] Oda Y., Wehrmann B., Radig K., Walter H., Rose I., Neumann W., Roessner A. (1995). Expression of growth factors and their receptors in human osteosarcomas. Immunohistochemical detection of epidermal growth factor, platelet-derived growth factor and their receptors: Its correlation with proliferating activities and p53 expression. Gen. Diagn. Pathol..

[22] Fromigue O., Hamidouche Z., Vaudin P., Lecanda F., Patino A., Barbry P., Mari B., Marie P.J. (2011). CYR61 downregulation reduces osteosarcoma cell invasion, migration, and metastasis. J. Bone Miner. Res..

[23] Hoang B.H., Kubo T., Healey J.H., Sowers R., Mazza B., Yang R., Huvos A.G., Meyers P.A., Gorlick R. (2004). Expression of LDL receptor-related protein 5 (LRP5) as a novel marker for disease progression in high-grade osteosarcoma. Int. J. Cancer.

[24] Boulytcheva I.V., Soloviev Y.N., Kushlinskii N.E., Mahson A.N. (2010). Expression of molecular markers in the tumor and survival prognosis in osteosarcoma. Bull. Exp. Biol. Med..

[25] Ferrari S., Bertoni F., Zanella L., Setola E., Bacchini P., Alberghini M., Versari M., Bacci G. (2004). Evaluation of P-glycoprotein, HER-2/ErbB-2, p53, and Bcl-2 in primary tumor and metachronous lung metastases in patients with high-grade osteosarcoma. Cancer.

[26] Gorlick R., Huvos A.G., Heller G., Aledo A., Beardsley G.P., Healey J.H., Meyers P.A. (1999). Expression of HER2/erbB-2 correlates with survival in osteosarcoma. J. Clin. Oncol..

[27] Onda M., Matsuda S., Higaki S., Iijima T., Fukushima J., Yokokura A., Kojima T., Horiuchi H., Kurokawa T., Yamamoto T. (1996). ErbB-2 expression is correlated with poor prognosis for patients with osteosarcoma. Cancer.

[28] Scotlandi K., Manara M.C., Hattinger C.M., Benini S., Perdichizzi S., Pasello M., Bacci G., Zanella L., Bertoni F., Picci P. (2005). Prognostic and therapeutic relevance of HER2 expression in osteosarcoma and Ewing's sarcoma. Eur. J. Cancer.

[29] Zhou H., Randall R.L., Brothman A.R., Maxwell T., Coffin C.M., Goldsby R.E. (2003). Her-2/neu expression in osteosarcoma increases risk of lung metastasis and can be associated with gene amplification. J. Pediatr. Hematol. Oncol..

[30] Perbal B., Zuntini M., Zambelli D., Serra M., Sciandra M., Cantiani L., Lucarelli E., Picci P., Scotlandi K. (2008). Prognostic value of CCN3 in osteosarcoma. Clin. Cancer Res..

[31] Bjornland K., Flatmark K., Pettersen S., Aaasen A.O., Fodstad O., Maelandsmo G.M. (2005). Matrix metalloproteinases participate in osteosarcoma invasion. J. Surg. Res..

[32] Gordon N., Koshkina N.V., Jia S.F., Khanna C., Mendoza A., Worth L.L., Kleinerman E.S. (2007). Corruption of the Fas pathway delays the pulmonary clearance of murine osteosarcoma cells, enhances their metastatic potential, and reduces the effect of aerosol gemcitabine. Clin. Cancer Res..

[33] Weber G.F., Bronson R.T., Ilagan J., Cantor H., Schmits R., Mak T.W. (2002). Absence of the CD44 gene prevents sarcoma metastasis. Cancer Res..

[34] Kubo T., Piperdi S., Rosenblum J., Antonescu C.R., Chen W., Kim H.S., Huvos A.G., Sowers R., Meyers P.A., Healey J.H. (2008). Platelet-derived growth factor receptor as a prognostic marker and a therapeutic target for imatinib mesylate therapy in osteosarcoma. Cancer.

[35] Sulzbacher I., Birner P., Trieb K., Traxler M., Lang S., Chott A. (2003). Expression of platelet-derived growth factor-AA is associated with tumor progression in osteosarcoma. Mod. Pathol..

[36] Mizobuchi H., Garcia-Castellano J.M., Philip S., Healey J.H., Gorlick R. (2008). Hypoxia markers in human osteosarcoma: An exploratory study. Clin. Orthop. Relat. Res..

[37] Kashima T., Nakamura K., Kawaguchi J., Takanashi M., Ishida T., Aburatani H., Kudo A., Fukayama M., Grigoriadis A.E. (2003). Overexpression of cadherins suppresses pulmonary metastasis of osteosarcoma *in vivo*. Int. J. Cancer.

[38] Ek E.T., Dass C.R., Contreras K.G., Choong P.F. (2007). PEDF-derived synthetic peptides exhibit antitumor activity in an orthotopic model of human osteosarcoma. J. Orthop. Res..

[39] Duan X., Jia S.F., Koshkina N., Kleinerman E.S. (2006). Intranasal interleukin-12 gene therapy enhanced the activity of ifosfamide against osteosarcoma lung metastases. Cancer.

[40] Xu J., Wu S., Shi X. (2010). Expression of matrix metalloproteinase regulator, RECK, and its clinical significance in osteosarcoma. J. Orthop. Res..

[41] Kaya M., Wada T., Nagoya S., Yamashita T. (2007). Prevention of postoperative progression of pulmonary metastases in osteosarcoma by antiangiogenic therapy using endostatin. J. Orthop. Sci..

[42] Luu H.H., Zhou L., Haydon R.C., Deyrup A.T., Montag A.G., Huo D., Heck R., Heizmann C.W., Peabody T.D., Simon M.A., He T.C. (2005). Increased expression of S100A6 is associated with decreased metastasis and inhibition of cell migration and anchorage independent growth in human osteosarcoma. Cancer Lett..

[43] Braczkowski R., Schally A.V., Plonowski A., Varga J.L., Groot K., Krupa M., Armatis P. (2002). Inhibition of proliferation in human MNNG/HOS osteosarcoma and SK-ES-1 Ewing sarcoma cell lines in vitro and in vivo by antagonists of growth hormone-releasing hormone: Effects on insulin-like growth factor II. Cancer.

[44] Burrow S., Andrulis I.L., Pollak M., Bell R.S. (1998). Expression of insulin-like growth factor receptor, IGF-1, and IGF-2 in primary and metastatic osteosarcoma. J. Surg. Oncol..

[45] Duan Z., Choy E., Harmon D., Yang C., Ryu K., Schwab J., Mankin H., Hornicek F.J. (2009). Insulin-like growth factor-I receptor tyrosine kinase inhibitor cyclolignan picropodophyllin inhibits proliferation and induces apoptosis in multidrug resistant osteosarcoma cell lines. Mol. Cancer Ther..

[46] Kolb E.A., Gorlick R., Houghton P.J., Morton C.L., Lock R., Carol H., Reynolds C.P., Maris J.M., Keir S.T., Billups C.A. (2008). Initial testing (stage 1) of a monoclonal antibody (SCH 717454) against the IGF-1 receptor by the pediatric preclinical testing program. Pediatr. Blood Cancer.

[47] Kolb E.A., Kamara D., Zhang W., Lin J., Hingorani P., Baker L., Houghton P., Gorlick R. (2010). R1507, a fully human monoclonal antibody targeting IGF-1R, is effective alone and in combination with rapamycin in inhibiting growth of osteosarcoma xenografts. Pediatr. Blood Cancer.

[48] Pinski J., Schally A.V., Groot K., Halmos G., Szepeshazi K., Zarandi M., Armatis P. (1995). Inhibition of growth of human osteosarcomas by antagonists of growth hormone-releasing hormone. J. Natl. Cancer Inst..

[49] Pinski J., Schally A.V., Halmos G., Szepeshazi K., Groot K. (1996). Somatostatin analog RC-160 inhibits the growth of human osteosarcomas in nude mice. Int. J. Cancer.

[50] Pollack R. (1990). Suramin Blockade of InsulinLike Growth Factor I-Stimulated Proliferation of Human Osteosarcoma Cells. J. Natl. Cancer Inst..

[51] Sekyi-Otu A., Bell R.S., Ohashi C., Pollak M., Andrulis I.L. (1995). Insulin-like growth factor 1 (IGF-1) receptors, IGF-1, and IGF-2 are expressed in primary human sarcomas. Cancer Res..

[52] Kim S.Y., Lee C.H., Midura B.V., Yeung C., Mendoza A., Hong S.H., Ren L., Wong D., Korz W., Merzouk A. (2008). Inhibition of the CXCR4/CXCL12 chemokine pathway reduces the development of murine pulmonary metastases. Clin. Exp. Metastasis.

[53] Laverdiere C., Hoang B.H., Yang R., Sowers R., Qin J., Meyers P.A., Huvos A.G., Healey J.H., Gorlick R. (2005). Messenger RNA expression levels of CXCR4 correlate with metastatic behavior and outcome in patients with osteosarcoma. Clin. Cancer Res..

[54] Lin F., Zheng S.E., Shen Z., Tang L.N., Chen P., Sun Y.J., Zhao H., Yao Y. (2011). Relationships between levels of CXCR4 and VEGF and blood-borne metastasis and survival in patients with osteosarcoma. Med. Oncol..

[55] Oda Y., Yamamoto H., Tamiya S., Matsuda S., Tanaka K., Yokoyama R., Iwamoto Y., Tsuneyoshi M. (2006). CXCR4 and VEGF expression in the primary site and the metastatic site of human osteosarcoma: Analysis within a group of patients, all of whom developed lung metastasis. Mod. Pathol..

[56] Dalla-Torre C.A., Yoshimoto M., Lee C.H., Joshua A.M., de Toledo S.R., Petrilli A.S., Andrade J.A., Chilton-MacNeill S., Zielenska M., Squire J.A. (2006). Effects of THBS3, SPARC and SPP1 expression on biological behavior and survival in patients with osteosarcoma. BMC Cancer.

[57] Engin F., Bertin T., Ma O., Jiang M.M., Wang L., Sutton R.E., Donehower L.A., Lee B. (2009). Notch signaling contributes to the pathogenesis of human osteosarcomas. Hum. Mol. Genet..

[58] Ferrari C., Benassi S., Ponticelli F., Gamberi G., Ragazzini P., Pazzaglia L., Balladelli A., Bertoni F., Picci P. (2004). Role of MMP-9 and its tissue inhibitor TIMP-1 in human osteosarcoma: Findings in 42 patients followed for 1–16 years. Acta Orthop. Scand..

[59] Yang C., Ji D., Weinstein E.J., Choy E., Hornicek F.J., Wood K.B., Liu X., Mankin H., Duan Z. (2010). The kinase Mirk is a potential therapeutic target in osteosarcoma. Carcinogenesis.

[60] Kersting C., Gebert C., Agelopoulos K., Schmidt H., van Diest P.J., Juergens H., Winkelmann W., Kevric M., Gosheger G., Brandt B. (2007). Epidermal growth factor receptor expression in high-grade osteosarcomas is associated with a good clinical outcome. Clin. Cancer Res..

[61] Dass C.R., Nadesapillai A.P., Robin D., Howard M.L., Fisher J.L., Zhou H., Choong P.F. (2005). Downregulation of uPAR confirms link in growth and metastasis of osteosarcoma. Clin. Exp. Metastasis.

[62] Uchibori M., Nishida Y., Nagasaka T., Yamada Y., Nakanishi K., Ishiguro N. (2006). Increased expression of membrane-type matrix metalloproteinase-1 is correlated with poor prognosis in patients with osteosarcoma. Int. J. Oncol..

[63] Khanna C., Wan X., Bose S., Cassaday R., Olomu O., Mendoza A., Yeung C., Gorlick R., Hewitt S.M., Helman L.J. (2004). The membrane-cytoskeleton linker ezrin is necessary for osteosarcoma metastasis. Nat. Med..

[64] Kim M.S., Song W.S., Cho W.H., Lee S.Y., Jeon D.G. (2007). Ezrin expression predicts survival in stage IIB osteosarcomas. Clin. Orthop. Relat. Res..

[65] Abdeen A., Chou A.J., Healey J.H., Khanna C., Osborne T.S., Hewitt S.M., Kim M., Wang D., Moody K., Gorlick R. (2009). Correlation between clinical outcome and growth factor pathway expression in osteogenic sarcoma. Cancer.

[66] Charity R.M., Foukas A.F., Deshmukh N.S., Grimer R.J. (2006). Vascular endothelial growth factor expression in osteosarcoma. Clin. Orthop. Relat. Res..

[67] Feng Y., Hu J., Ma J., Feng K., Zhang X., Yang S., Wang W., Zhang J., Zhang Y. (2011). RNAi-mediated silencing of VEGF-C inhibits non-small cell lung cancer progression by simultaneously down-regulating the CXCR4, CCR7, VEGFR-2 and VEGFR-3-dependent axes-induced ERK, p38 and AKT signalling pathways. Eur. J. Cancer.

[68] Kaya M., Wada T., Akatsuka T., Kawaguchi S., Nagoya S., Shindoh M., Higashino F., Mezawa F., Okada F., Ishii S. (2000). Vascular endothelial growth factor expression in untreated osteosarcoma is predictive of pulmonary metastasis and poor prognosis. Clin. Cancer Res..

[69] Kaya M., Wada T., Kawaguchi S., Nagoya S., Yamashita T., Abe Y., Hiraga H., Isu K., Shindoh M., Higashino F. (2002). Increased pre-therapeutic serum vascular endothelial growth factor in patients with early clinical relapse of osteosarcoma. Br. J. Cancer.

[70] Kaya M., Wada T., Nagoya S., Sasaki M., Matsumura T., Yamashita T. (2009). The level of vascular endothelial growth factor as a predictor of a poor prognosis in osteosarcoma. J. Bone Joint Surg. Br..

[71] Lee Y.H., Tokunaga T., Oshika Y., Suto R., Yanagisawa K., Tomisawa M., Fukuda H., Nakano H., Abe S., Tateishi A. (1999). Cell-retained isoforms of vascular endothelial growth factor (VEGF) are correlated with poor prognosis in osteosarcoma. Eur. J. Cancer.

[72] Sulzbacher I., Birner P., Trieb K., Lang S., Chott A. (2002). Expression of osteopontin and vascular endothelial growth factor in benign and malignant bone tumors. Virchows Arch..

[73] Foukas A.F., Deshmukh N.S., Grimer R.J., Mangham D.C., Mangos E.G., Taylor S. (2002). Stage-IIB osteosarcomas around the knee. A study of MMP-9 in surviving tumour cells. J. Bone Joint Surg. Br..

[74] Himelstein B.P., Asada N., Carlton M.R., Collins M.H. (1998). Matrix metalloproteinase-9 (MMP-9) expression in childhood osseous osteosarcoma. Med. Pediatr. Oncol..

[75] Gordon N., Arndt C.A., Hawkins D.S., Doherty D.K., Inwards C.Y., Munsell M.F., Stewart J., Koshkina N.V., Kleinerman E.S. (2005). Fas expression in lung metastasis from osteosarcoma patients. J. Pediatr. Hematol. Oncol..

[76] Koshkina N.V., Khanna C., Mendoza A., Guan H., DeLauter L., Kleinerman E.S. (2007). Fas-negative osteosarcoma tumor cells are selected during metastasis to the lungs: The role of the Fas pathway in the metastatic process of osteosarcoma. Mol. Cancer Res..

[77] Lafleur E.A., Koshkina N.V., Stewart J., Jia S.F., Worth L.L., Duan X., Kleinerman E.S. (2004). Increased Fas expression reduces the metastatic potential of human osteosarcoma cells. Clin Cancer Res..

[78] Asai T., Tomita Y., Nakatsuka S., Hoshida Y., Myoui A., Yoshikawa H., Aozasa K. (2002). VCP (p97) regulates NFkappaB signaling pathway, which is important for metastasis of osteosarcoma cell line. Jpn. J. Cancer Res..

[79] Rubin E.M., Guo Y., Tu K., Xie J., Zi X., Hoang B.H. (2010). Wnt inhibitory factor 1 decreases tumorigenesis and metastasis in osteosarcoma. Mol. Cancer Ther..

[80] De Nigris F., Rossiello R., Schiano C., Arra C., Williams-Ignarro S., Barbieri A., Lanza A., Balestrieri A., Giuliano M.T., Ignarro L.J. (2008). Deletion of Yin Yang 1 protein in osteosarcoma cells on cell invasion and CXCR4/angiogenesis and metastasis. Cancer Res..

[81] Han J.D., Bertin N., Hao T., Goldberg D.S., Berriz G.F., Zhang L.V., Dupuy D., Walhout A.J., Cusick M.E., Roth F.P. (2004). Evidence for dynamically organized modularity in the yeast protein-protein interaction network. Nature.

[82] Aziz M.H., Manoharan H.T., Verma A.K. (2007). Protein kinase C epsilon, which sensitizes skin to sun’s UV radiation-induced cutaneous damage and development of squamous cell carcinomas, associates with Stat3. Cancer Res..

[83] Gundimeda U., Schiffman J.E., Gottlieb S.N., Roth B.I., Gopalakrishna R. (2009). Negation of the cancer-preventive actions of selenium by over-expression of protein kinase Cepsilon and selenoprotein thioredoxin reductase. Carcinogenesis.

[84] Hafeez B.B., Zhong W., Weichert J., Dreckschmidt N.E., Jamal M.S., Verma A.K. (2011). Genetic ablation of PKC epsilon inhibits prostate cancer development and metastasis in transgenic mouse model of prostate adenocarcinoma. Cancer Res..

[85] McJilton M.A., van Sikes C., Wescott G.G., Wu D., Foreman T.L., Gregory C.W., Weidner D.A., Harris Ford O., Morgan Lasater A., Mohler J.L. (2003). Protein kinase Cepsilon interacts with Bax and promotes survival of human prostate cancer cells. Oncogene.

[86] Meshki J., Caino M.C., von Burstin V.A., Griner E., Kazanietz M.G. (2010). Regulation of prostate cancer cell survival by protein kinase Cepsilon involves bad phosphorylation and modulation of the TNFalpha/JNK pathway. J. Biol. Chem..

[87] Wu D., Foreman T.L., Gregory C.W., McJilton M.A., Wescott G.G., Ford O.H., Alvey R.F., Mohler J.L., Terrian D.M. (2002). Protein kinase cepsilon has the potential to advance the recurrence of human prostate cancer. Cancer Res..

[88] Grossoni V.C., Todaro L.B., Kazanietz M.G., de Kier Joffe E.D., Urtreger A.J. (2009). Opposite effects of protein kinase C beta1 (PKCbeta1) and PKCepsilon in the metastatic potential of a breast cancer murine model. Breast Cancer Res. Treat..

[89] Pan Q., Bao L.W., Kleer C.G., Sabel M.S., Griffith K.A., Teknos T.N., Merajver S.D. (2005). Protein kinase C epsilon is a predictive biomarker of aggressive breast cancer and a validated target for RNA interference anticancer therapy. Cancer Res..

[90] Huang B., Cao K., Li X., Guo S., Mao X., Wang Z., Zhuang J., Pan J., Mo C., Chen J. (2011). The expression and role of protein kinase C (PKC) epsilon in clear cell renal cell carcinoma. J. Exp. Clin. Cancer Res..

[91] Jansen A.P., Verwiebe E.G., Dreckschmidt N.E., Wheeler D.L., Oberley T.D., Verma A.K. (2001). Protein kinase C-epsilon transgenic mice: A unique model for metastatic squamous cell carcinoma. Cancer Res..

[92] Reddig P.J., Dreckschmidt N.E., Zou J., Bourguignon S.E., Oberley T.D., Verma A.K. (2000). Transgenic mice overexpressing protein kinase C epsilon in their epidermis exhibit reduced papilloma burden but enhanced carcinoma formation after tumor promotion. Cancer Res..

[93] Sand J.M., Aziz M.H., Dreckschmidt N.E., Havighurst T.C., Kim K., Oberley T.D., Verma A.K. (2010). PKCepsilon overexpression, irrespective of genetic background, sensitizes skin to UVR-induced development of squamous-cell carcinomas. J. Invest. Dermatol..

[94] Sand J.M., Hafeez B.B., Aziz M.H., Siebers E.M., Dreckschmidt N.E., Verma A.K. (2012). Ultraviolet radiation and 12-*O*-tetradecanoylphorbol-13-acetate-induced interaction of mouse epidermal protein kinase C epsilon with Stat3 involve integration with Erk1/2. Mol. Carcinog..

[95] Wheeler D.L., Ness K.J., Oberley T.D., Verma A.K. (2003). Inhibition of the development of metastatic squamous cell carcinoma in protein kinase C epsilon transgenic mice by alpha-difluoromethylornithine accompanied by marked hair follicle degeneration and hair loss. Cancer Res..

[96] Bao L., Gorin M.A., Zhang M., Ventura A.C., Pomerantz W.C., Merajver S.D., Teknos T.N., Mapp A.K., Pan Q. (2009). Preclinical development of a bifunctional cancer cell homing, PKCepsilon inhibitory peptide for the treatment of head and neck cancer. Cancer Res..

[97] Martinez-Gimeno C., Diaz-Meco M.T., Dominguez I., Moscat J. (1995). Alterations in levels of different protein kinase C isotypes and their influence on behavior of squamous cell carcinoma of the oral cavity: Epsilon PKC, a novel prognostic factor for relapse and survival. Head Neck.

[98] Pan Q., Bao L.W., Teknos T.N., Merajver S.D. (2006). Targeted disruption of protein kinase C epsilon reduces cell invasion and motility through inactivation of RhoA and RhoC GTPases in head and neck squamous cell carcinoma. Cancer Res..

[99] Tosi L., Rinaldi E., Carinci F., Farina A., Pastore A., Pelucchi S., Cassano L., Evangelisti R., Carinci P., Volinia S. (2005). Akt, protein kinase C, and mitogen-activated protein kinase phosphorylation status in head and neck squamous cell carcinoma. Head Neck.

[100] Bae K.M., Wang H., Jiang G., Chen M.G., Lu L., Xiao L. (2007). Protein kinase C epsilon is overexpressed in primary human non-small cell lung cancers and functionally required for proliferation of non-small cell lung cancer cells in a p21/Cip1-dependent manner. Cancer Res..

[101] Caino M.C., von Burstin V.A., Lopez-Haber C., Kazanietz M.G. (2011). Differential regulation of gene expression by protein kinase C isozymes as determined by genome-wide expression analysis. J. Biol. Chem..

[102] Ding L., Wang H., Lang W., Xiao L. (2002). Protein kinase C-epsilon promotes survival of lung cancer cells by suppressing apoptosis through dysregulation of the mitochondrial caspase pathway. J. Biol. Chem..

[103] Felber M., Sonnemann J., Beck J.F. (2007). Inhibition of novel protein kinase *C*-epsilon augments TRAIL-induced cell death in A549 lung cancer cells. Pathol. Oncol. Res..

[104] Aviel-Ronen S., Coe B.P., Lau S.K., da Cunha Santos G., Zhu C.Q., Strumpf D., Jurisica I., Lam W.L., Tsao M.S. (2008). Genomic markers for malignant progression in pulmonary adenocarcinoma with bronchioloalveolar features. Proc. Natl. Acad. Sci. USA.

[105] Yang D., Kedei N., Li L., Tao J., Velasquez J.F., Michalowski A.M., Toth B.I., Marincsak R., Varga A., Biro T. (2010). RasGRP3 contributes to formation and maintenance of the prostate cancer phenotype. Cancer Res..

[106] Kimura K., Nakano T., Park Y.B., Tani M., Tsuda H., Beppu Y., Moriya H., Yokota J. (2002). Establishment of human osteosarcoma cell lines with high metastatic potential to lungs and their utilities for therapeutic studies on metastatic osteosarcoma. Clin. Exp. Metastasis.

[107] Nakano T., Tani M., Ishibashi Y., Kimura K., Park Y.B., Imaizumi N., Tsuda H., Aoyagi K., Sasaki H., Ohwada S. (2003). Biological properties and gene expression associated with metastatic potential of human osteosarcoma. Clin. Exp. Metastasis.

[108] Jia S.F., Worth L.L., Kleinerman E.S. (1999). A nude mouse model of human osteosarcoma lung metastases for evaluating new therapeutic strategies. Clin. Exp. Metastasis.

[109] Zhu W., Guo F., Xu T., Chen A. (2007). Establishment of nude mice model of human osteosarcoma cell line MG63 with different potential of metastasis. Chin. Ger. J. Clin. Oncol..

[110] England K., Ashford D., Kidd D., Rumsby M. (2002). PKC epsilon is associated with myosin IIA and actin in fibroblasts. Cell. Signal..

[111] Allen T.R., Krueger K.D., Hunter K.W., Agrawal D.K. (2005). Evidence that insulin-like growth factor-1 requires protein kinase C-(epsilon), PI3-kinase and mitogen-activated protein kinase pathways to protect human vascular smooth muscle cells from apoptosis. Immunol. Cell. Biol..

[112] Thommes K.B., Hoppe J., Vetter H., Sachinidis A. (1996). The synergistic effect of PDGF-AA and IGF-1 on VSMC proliferation might be explained by the differential activation of their intracellular signaling pathways. Exp. Cell Res..

[113] Yano K., Bauchat J.R., Liimatta M.B., Clemmons D.R., Duan C. (1999). Down-regulation of protein kinase C inhibits insulin-like growth factor I-induced vascular smooth muscle cell proliferation, migration, and gene expression. Endocrinology.

[114] Newton A.C. (2003). Regulation of the ABC kinases by phosphorylation: Protein kinase C as a paradigm. Biochem. J..

[115] Mamane Y., Petroulakis E., LeBacquer O., Sonenberg N. (2006). mTOR, translation initiation and cancer. Oncogene.

[116] Hua Y., Qiu Y., Zhao A., Wang X., Chen T., Zhang Z., Chi Y., Li Q., Sun W., Li G. (2011). Dynamic metabolic transformation in tumor invasion and metastasis in mice with LM-8 osteosarcoma cell transplantation. J. Proteome Res..

[117] Huang B., Cao K., Li X., Guo S., Mao X., Wang Z., Zhuang J., Pan J., Mo C., Chen J. (2011). The expression and role of protein kinase C (PKC) epsilon in clear cell renal cell carcinoma. J. Exp. Clin. Cancer Res..

[118] Leask A., Shi-Wen X., Khan K., Chen Y., Holmes A., Eastwood M., Denton C.P., Black C.M., Abraham D.J. (2008). Loss of protein kinase Cepsilon results in impaired cutaneous wound closure and myofibroblast function. J. Cell. Sci..

[119] Simon R.M., Lam A., Li M.C., Ngan M., Menenzes S., Zhao Y. (2007). Analysis of Gene Expression Data Using BRB-Array Tools. Cancer Inform..

[120] Merico D., Isserlin R., Stueker O., Emili A., Bader G.D. (2010). Enrichment map: A network-based method for gene-set enrichment visualization and interpretation. PLoS One.

[121] Cline M.S., Smoot M., Cerami E., Kuchinsky A., Landys N., Workman C., Christmas R., Avila-Campilo I., Creech M., Gross B. (2007). Integration of biological networks and gene expression data using Cytoscape. Nat. Protoc..

[122] Harris M.A., Clark J., Ireland A., Lomax J., Ashburner M., Foulger R., Eilbeck K., Lewis S., Marshall B., Mungall C. (2004). The Gene Ontology (GO) database and informatics resource. Nucleic Acids Res..

[123] Boyle E.I., Weng S., Gollub J., Jin H., Botstein D., Cherry J.M., Sherlock G. (2004). GO::TermFinder—open source software for accessing Gene Ontology information and finding significantly enriched Gene Ontology terms associated with a list of genes. Bioinformatics.

[124] Eppert K., Wunder J.S., Aneliunas V., Kandel R., Andrulis I.L. (2005). von Willebrand factor expression in osteosarcoma metastasis. Mod. Pathol..

[125] Gorin M.A., Pan Q. (2009). Protein kinase C epsilon: An oncogene and emerging tumor biomarker. Mol. Cancer.

[126] Griner E.M., Kazanietz M.G. (2007). Protein kinase C and other diacylglycerol effectors in cancer. Nat. Rev. Cancer.

